# Identifying predictors of blood pressure control in the Lebanese population - a national, multicentric survey – I-PREDICT

**DOI:** 10.1186/1471-2458-14-1142

**Published:** 2014-11-05

**Authors:** Samir G Mallat, Serena Abu Samra, Fariha Younes, Marie-Thérèse Sawaya

**Affiliations:** Department of Internal Medicine, American University of Beirut, Riad El-Solh, P.O. Box 11–0236, Beirut, 11072020 Lebanon; Medical Affairs Department, Sanofi-aventis Liban, Omar Daouk Street, Starco Building, 1st Floor, Bloc B, P.O. Box 110697, Beirut, Lebanon

**Keywords:** Anti-hypertensive drugs, Blood pressure, Control, Guidelines, Predictors

## Abstract

**Background:**

Blood Pressure (BP) is not well controlled and factors that predict BP control are not well identified in Lebanon. Improvement of hypertension management requires an understanding of patients’ characteristics and factors associated with uncontrolled BP. This national, multicentric, observational prospective study was designed to determine the predictors of BP control in patients followed up to 6 months.

**Methods:**

I-PREDICT study was conducted on 988 patients with newly diagnosed or uncontrolled hypertension. Socio-demographic and clinical characteristics were analyzed. The level of agreement between doctors’ perceptions on BP control status and JNC VII guidelines was analyzed.

**Results:**

The predictor associated with poor BP control was diabetes (OR = 0.17, CI = 0.10–0.28 at month-1; OR = 0.15, CI = 0.10–0.24 at month-6). The predictors associated with better BP control at month-6 were the early control of BP at month-1 (OR = 10.39, CI = 6.18–17.47) and combination therapy prescribed at baseline and month-1 (OR = 15.14, CI = 1.09–208.46, P = 0.04). In the sub-group of diabetes, the predictors that were associated with better BP control at 6 months were following diet at V1 (OR = 2.27, CI = 1.01 to 5.12) and BP control at V2 (OR = 7.34, CT = 3.83 to 14.07). The predictors that were associated with poor BP control at 6 months were middle economic class (OR = 0.036, CI = 0.16-0.94) and upper economic class (OR = 0.036; CI = 0.13-0.93).

The rate of BP control was significantly higher at month 6 *versus* month 1 (67.52% *vs* 44.08%, *P* = 0.001). Additional analysis showed poor agreement between the doctors’ perceptions on BP control status and the guidelines.

**Conclusions:**

Reaching an early BP control and combination therapy were significant predictors of better BP control, whereas diabetes was a significant predictor of poor BP control. A poor agreement between JNC VII guidelines and clinical practice was observed. I-PREDICT study identified factors that can be targeted for improving BP control.

## Background

High blood pressure is a major risk factor for cardiovascular diseases, it is estimated to account for 13% of deaths worldwide [1]. Deaths from stroke in the Middle East and North Africa will nearly double by 2030 [2]. Furthermore, almost three-quarters of people with hypertension (639 million people) live in developing countries with limited health resources and where people have a very low awareness of hypertension and poor blood pressure control [1, 3]. Within the past few years, the prevalence of hypertension is increasing and is predicted to grow by more than 500 million by 2025 [4, 5].

In developing countries including Lebanon, the high prevalence of hypertension and poor hypertension control are important factors in the rising epidemic of cardiovascular disease. In Lebanon, around 39% prevalence of hypertension is reported among the general population [6]. Lebanon has a higher prevalence of hypertension compared to Tunisia (30, 6%) [7], Greece (30, 5%) [8], Turkey (31.8%) [9] and Jordan (15%) [10]. Among patients with coronary artery disease studied in The Lebanese Interventional Coronary Registry (LICOR) in 2011, the most common reported cardiovascular risk factor was high BP reaching a prevalence of around 62.5% [11]. LICOR is in a national registry of more than 47 092 interventional events by 2011 (cardiac catheterization and percutaneous coronary intervention) from more than 40 sites across all Lebanon. These results highlight the urgent need for intervention to prevent, treat and control hypertension [12].

The main challenge about hypertension is the number of patients who are not in control of their BP. If left uncontrolled, hypertension can lead to coronary artery disease, an enlargement of the heart and eventually heart failure [13]. The number of uncontrolled hypertensive patients also varies by countries [14]. Such that, in USA, analysis of data from the National Health and Nutrition Examination Survey (NHANES) 2003–2010 showed that among patients with hypertension in the general population, an estimated 35.8 million (53.5%) did not have their hypertension controlled at the 140/90 mm Hg threshold [15]. Meanwhile, within Europe, rates of BP control among hypertensive treated cases were 40%, 30%, 28%, 19% and 21% respectively in England, Germany, Italy, Spain and Sweden at the 140/90 mm Hg threshold from 1997 to 1990 [14]. Similarly, hypertension was not controlled to the recommended levels of BP in about one-half (50.4%) of patients during the period of June to December 2006 in Jordan.

Globally, the low BP control rates have been difficult to explain, given the number of apparently well-tolerated medications available for the management of hypertension. Factors related to access to care, medical practice patterns, patient education, poor compliance to prescribed treatment and patient cardio-metabolic profile have all been proposed as barriers to BP control [16–18]. However, BP is also determined by other factors, including age, severity of disease, health habits, and early control and co-morbidities [17, 18].

Further identification of patients at risk of poor control can lead to targeted interventions to improve management of hypertension. Besides, in order to improve the BP control rate in the population with hypertension, a more complete understanding of the predictors of BP control is essential.

The following study (I-PREDICT) is a national, multicentric, observational, prospective cohort survey which aimed to identify the predictors of BP control in hypertensive Lebanese patients followed up to 6 months. The secondary objectives were to identify the predictors after 1 month of follow-up and to evaluate the number of hypertensive patients who are and are not at goal at months 1 and 6.

## Subjects and methods

### Study population

From April 2010 to November 2010, a national, multicentric, observational, prospective cohort survey was conducted on 1079 patients, with newly diagnosed or uncontrolled hypertension on previous medications, in 107 centers all over Lebanon. Participating centers were either private clinics or hospitals based outpatient clinics.

Institutional review board (Makassed General Hospital and Rafic Hariri University Hospital) approved the study protocol and the study was performed in compliance with Good Clinical Practices.

## Methods

One hundred seven (107) randomly selected physicians from across Lebanon were invited to participate in the study. Their number was determined based on the sample size and the recruitment period. Each physician was requested to enroll the first 10 eligible patients who met the eligibility criteria and gave written informed consent. Eligible participants for this survey were men and women 18 years old or above with newly diagnosed hypertension or uncontrolled hypertension on medications. Exclusion criteria included pregnancy, lactation, secondary hypertension or participation in another study.

Hypertension was considered uncontrolled if BP was more than 140/90 mm Hg in hypertensive patients and more than 130/80 mm Hg in hypertensive patients with diabetes and chronic kidney disease. Those thresholds were based on the latest report of the Joint National Committee on Prevention, Detection, Evaluation, and Treatment of High Blood Pressure (JNC VII) and the guidelines for the management of arterial hypertension of the European Society of Hypertension [19, 20].

### Data collection

Case report forms in English were used to collect patients’ data, from the physicians, over 6 months follow-up after enrolment in the study. Data were collected at baseline and during two follow-up visits when performed by the patients. The information included the following: dates of visits, verification of the eligibility criteria, systolic and diastolic blood pressures, whether the patient is newly diagnosed with hypertension or not, co-morbidities (diabetes, atherosclerotic cardiovascular diseases, dyslipidemia and chronic kidney disease), lifestyle (diet, exercise and smoking), medical treatment for hypertension and number of prescribed antihypertensive medication. The survey also obtained data on patient’s gender, weight, height, waist circumference, educational level, marital and socio-economic status assessed by the investigator, home blood pressure monitor (possession of any validated sphygmomanometer at home) and knowledge of the patient of his target blood pressure.

This study is purely observational of real-life practices, therefore no specific method of evaluation was defined. Physician’s therapeutic decisions and patients’ assessment were based on usual real-life practices.

BP was measured by the available blood pressure device. Although, we do realize that using an automatic device is an objective method of BP assessment but in line with the study design, we didn’t impose any specific tool in order not to introduce any bias in the “real-life” assessment.

Reported diabetes was based on a history of diabetes mellitus as diagnosed by a physician or other health professional, or if they were receiving insulin or oral diabetic medications. Diabetes type 1 and 2 were analyzed together. Educational level was defined as the highest level of education completed. Based on the physician’s assessment of patients’ living conditions and satisfaction of needs, as done in real-life, the socio-economic status was classified as being low, middle or high. Waist circumference was measured to the nearest centimeter using non-stretchable tailors measuring tape at the midway between the lower rib margin and the iliac crest. The patient was asked to breathe normally and the reading was taken at the end of the light exhale. Height and weight were measured in patients wearing light indoor clothes and no shoes. Answers for these two parameters were used to calculate the body mass index (BMI) of each patient as weight (in kilograms, kg) divided by height (in meters, m) squared (kg/m^2^). Normal weight was defined as having a BMI of 18.5–24.9 kg/m^2^, overweight of 25.0–29.9 kg/m^2^ and obesity as BMI ≥ 30 kg/m^2^, as defined by the World Health Organization (WHO) classification of 2004 [21].

### Statistical analysis

Sample descriptive statistics, including frequencies, percentages, means, standard deviations and ranges, were calculated to summarize the socio-demographic and clinical characteristics of the study cohort at baseline. Continuous variables were expressed as means and standard deviations and categorical variables as percentages.

A multivariate analysis using logistic regression models was conducted to identify independent predictors of BP control after 1 month and 6 months of follow-up. The initial full models with BP control as the outcome variable included the following set of covariates/predictor variables: diabetes, education level, following diet, myocardial infarction, dyslipidemia, atrial fibrillation, chronic kidney disease, smoker, exercising, newly diagnosed, combination therapy at baseline, months 1 and 6, and BP controlled at months 1 and 6. The risks were reported as odds ratios (OR) with corresponding 95% confidence intervals (CI). A two-tailed P value <0.05 was considered statistically significant.

Besides, the statistical analysis evaluated the number of hypertensive patients who were or not at goal at months 1 and 6. Chi-square test was used for categorical outcomes (controlled/uncontrolled BP) using a two-tailed P value <0.05 for statistical significance.

Importantly, additional analysis was performed to assess the level of agreement between doctors’ perceptions on blood pressure control status and JNC VII guidelines Indeed, Physicians were asked about the categorization of their patients’ blood pressure status. According to JNC7 Guidelines, patients’ blood pressure control status was identified separately. Using SPSS, Kappa agreement according to Landis and Kosh’s classification, which is universally accepted (Table 1) [22] was calculated between the two variables. The statistical analysis was carried out using STATA and SPSS softwares for Windows Release (Stata Corp, College Station, Texas, USA, version 11 and SPSS Inc., Chicago, USA, version 17.0).

**Table 1:** **Interpretation of kappa according to Landis and Kosh** [22]

Kappa	Agreement
< 0	Less than chance agreement (poor)
0.01–0.20	Slight agreement
0.21–0.40	Fair agreement
0.41–0.60	Moderate agreement
0.61–0.80	Substantial agreement
0.81–0.99	Almost perfect agreement

## Results

### Recruitment

**Figure 1:**
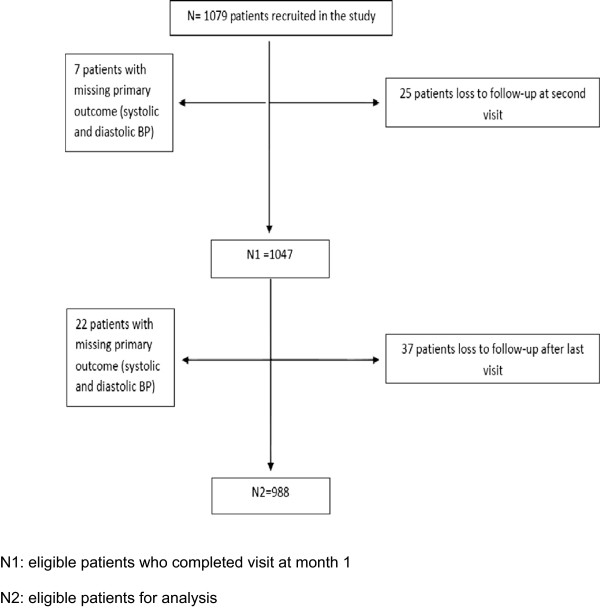
**Patients’ flowchart.**

A total of 1079 patients in 107 centers all over Lebanon were recruited in this survey of whom 988 were eligible for the statistical analysis (Figure 1).

### Socio-demographic characteristics of the patients at baseline

Overall, 55% (N = 543) of the patients were men and 45% (N = 445) were women. The percentages of patients with newly diagnosed hypertension *versus* known to be hypertensive on medications were respectively 51.3% and 48.7%.

At baseline, out of 988 patients, the mean systolic BP was 166.74 ± 37.74 mm Hg, and the mean diastolic BP was 94.01 ± 9.18 mm Hg. The mean BMI was 28.75 ± 4.08 Kg/m^2^. The average waist circumference was 98.42 ± 16.02 cm.

Table 2 presents basic socio-demographic information of the cohort study all over Lebanon.

**Table 2:** **Socio-demographic information of the cohort study at baseline**

	% N = 988
Gender	
Female	45.04%
Male	54.96%
House location	
Urban	66.80%
Rural	33.20%
Economic class	
Low	10.25%
Middle	70.20%
High	19.55%
Marital status	
Married	81.02%
Single	11.94%
Divorced	2.25%
Widow	4.79%
Patients’ education level	
Illiterate	5.22%
Primary school	35.69%
Secondary school	36.89%
Higher education	22.20%

### Sub-group analysis of the diabetic population

There is a significant difference in the geographic distribution of diabetic versus non-diabetic patients such that patients in Beirut are more likely to be non-diabetic (p = 0.002). Additionally there is a significant difference in the educational levels of diabetic patients versus non-diabetic patients where non-diabetic patients are more likely to have higher educational levels (p = 0.022). Details are shown in Table 3.

**Table 3:** **Comparison of socio-demographic information of between diabetic and non-diabetic populations**

		Diabetes	No diabetes	P-value
Gender	**Males**	199	356	0.1
	**Females**	182	281	
Districts	**Beirut**	162	205	0.002
	**North**	42	77	
	**South**	39	100	
	**Bekaa**	18	49	
	**Mount Leb**	110	175	
Economic status	**Lower**	45	54	0.567
	**Middle**	249	436	
	**Upper**	72	118	
Highest completed education	**Illiterate**	27	21	0.022
	**Primary school**	135	203	
	**Secondary school**	122	229	
	**hHigher education**	73	140	

### Clinical characteristics of the patients at baseline

Regarding co-morbidities, 41.27% were smokers. Dyslipidemia and diabetes were more frequently present with percentages of 57.39% and 39.27% respectively. Co-morbidities of patients at baseline are detailed in Table 4.

**Table 4:** **Co-morbidities of patients at baseline**

Co-morbidity	% N = 988
Dyslipidemia	57.39%
Smokers	41.27%
Diabetes	39.27%
Obesity	34.5%
Left ventricular hypertrophy	26.31%
Myocardial infarction	12.22%
Chronic kidney disease	4.62%
Congestive Heart failure	4.12%
Atrial fibrillation	3.90%
Stroke	3.49%

There is no significant difference between diabetic versus non-diabetic patients; such that the mean systolic and diastolic blood pressure, weight and waist circumference were comparable between the two subgroups.

### Distribution of anti-hypertensive medications

At baseline, the majority of patients received a combination therapy (71.3% vs. 28.7% on monotherapy) and this observation was maintained same across visits. Details on distribution of anti-hypertensive medication is detailed in Table 5.

**Table 5:** **Distribution of anti-hypertensive medication per visit**

Variables		Distribution at baseline (%)	Distribution at V2 (%)	Distribution at V3 (%)
Monotherapy	Diuretic	0.2	0.2	0.31
	Beta blocker	0.5	0.51	0.1
	ARB	26.5	26.44	22.85
	ACEI	0.9	0.91	0.72
	CCB	0.6	0.61	0.31
Combination		71.3	71.33	75.71

*ARB* = Angiotensin Receptor Blocker; *ACEI* = Angiotensin Converting Enzyme; Inhibitor; *CCB* = Calcium Channel Blocker.

### Predictors of BP control

In multivariate analysis, a number of factors were found to be associated with BP control in hypertensive patients at the follow-up visits (months 1 and 6) (Table 6).

**Table 6:** **Predictors associated with blood pressure control in multivariate analysis (follow-up visit at month 6)**

Covariates	OR	95% CI	P value
Diabetes	0.15	0.10–0.24	0.00*
BP control at month 1	10.39	6.18-17.47	0.00*
Combination therapy at baseline and month 1	15.14	1.09–208.46	0.04*
Educational level			
Primary school	1.07	0.29–4.00	0.92
Secondary school	0.73	0.19–2.70	0.63
Higher education	0.81	0.19–3.31	0.77
Following diet	1.30	0.68–2.42	0.42
Myocardial infarction	0.71	0.40–1.26	0.24
Dyslipidemia	1.34	0.87–2.06	0.17
Atrial fibrillation	0.39	0.14–1.09	0.07
Chronic kidney disease	0.62	0.25–1.55	0.30
Smoker	1.07	0.72–1.59	0.72
Follow exercise	1.01	0.64–1.61	0.95
Newly diagnosed	0.87	0.57–1.34	0.54
Sub-group of diabetes			
Following diet at V1	2.27	1.01-5.12	0.047*
BP controlled at V2	7.34	3.83-14.07	0.000*
Middle economic class	0.39	0.16-0.94	0.036*
Upper middle class	0.30	0.13-0.93	0.036*

*CI* = Confidence interval; *OR* = Odds ratio.

*Significant.

The predictor that was associated with poor BP control at the two follow-up visits was diabetes (OR = 0.17, CI = 0.10 to 0.28 at month 1; OR = 0.15, CI = 0.10 to 0.24 at month 6).

The predictors that were associated with better BP control at month 6 were the early control of blood pressure at month 1 (OR = 10.39, CI = 6.18 to 17.47) and combination therapy at baseline and month1 (OR = 15.14, CI = 1.09 to 208.46, P = 0.04). Patients treated with combination therapy at baseline and month 1 had a better BP control at month 6 compared to those being treated with monotherapy at baseline.

No significant association with blood pressure control was found for educational level, following diet, exercising, smoking, dyslipidemia, myocardial infarction, atrial fibrillation, chronic kidney disease and new diagnosis of hypertension.

In the sub-group of diabetes, the predictors that were associated with better BP control at 6 months were following diet at V1 (OR = 2.27, CI = 1.01 to 5.12) and BP control at V2 (OR = 7.34, CT = 3.83 to 14.07). The predictors that were associated with poor BP control at 6 months were middle economic class (OR = 0.036, CI = 0.16-0.94) and upper economic class (OR = 0.036; CI = 0.13-0.93).

### Rate of BP control

The percentages of patients who had controlled blood pressure at months 1 and 6 were respectively 44.08% and 67.52%. The rate of BP control was significantly higher at month 6 *versus* month 1 (P = 0.00).

### Level of agreement between doctors’ perceptions on blood pressure status and JNC VII guidelines

The measurement of agreement between doctors’ perceptions on BP control and JNC VII guidelines showed a poor agreement between the two parameters at the follow-up visit at month 1 (kappa < 0) whereas it was fair at the follow-up visit at month 6 (kappa = 0.26) according to Landis and Koch characterization’s [22].

## Discussion

I-PREDICT survey is the first study that attempts to identify the predictors of BP control in the Lebanese population nationwide. It identifies diabetes as a predictor of poor BP control in subjects with hypertension. This is consistent with findings from previous studies that showed a higher predilection for uncontrolled BP in diabetic patients [17, 23–26]. Despite the known fact that the prevalence of hypertension in adults with diabetes is very high [18, 27–29], results showed that diabetic patients with hypertension are poorly controlled as compared to non-diabetic patients [18, 27]. These findings highlight a distinct risk in the diabetic population since diabetes is associated with high cardiovascular mortality. Indeed, the Framingham study has revealed that much of this risk is due to coexisting hypertension [30]. Both macrovascular and microvascular complications are increased in hypertensive diabetic patients when compared to normotensive diabetic patients [26]. In addition, hypertension was the strongest determinant of cardiovascular outcomes in diabetic patients [30]. Since the diabetic population represents a significant percentage of the general population in Lebanon (7-8% nationwide [31] v/s 15.8% in Great Beirut [32]), analysis of the predictors of BP control in this group would potentially help improve cardiovascular outcomes and mortality.

Moreover, early control of blood pressure is known to improve BP to a significant extent over time [18, 33, 34]. In fact, the multivariate analysis in our study also showed that early control of BP was a significant predictor for a better BP control at a later follow-up.

This study also showed that combination therapy had a statistical advantage in terms of BP control (OR = 15.14, CI = 1.09 to 208.46, P = 0.04). Patients who stayed on combination therapy by the time of the third follow-up visit at month 6 had a better BP control when they were already taking a combination therapy at baseline and month 1 as compared to those being treated with monotherapy at baseline. Several studies have shown that combination therapy may improve control rates of BP [35–37]. In addition, Egan et al. reported that initial antihypertensive combination therapy produced more rapid BP control than initial monotherapy in clinical trials [36].

The results of the study show also that the study population tends to be overweight as the mean BMI was 28.75 ± 4.08 kg/m^2^. Also, almost near 4 in 10 patients were smokers. These findings display the importance of pressing the need for lifestyle modification in hypertensive patients in Lebanon, since smoking abstinence and weight loss have shown to be key players in BP control [20].

Follow-up results showed a significantly larger population reaching control at 6 month (67.52%) as compared to month 1 (44.08%). The overall BP control rate, after 6 months, in this cohort, is higher than international studies’ rates [15, 38, 39]. Such that, 50% of the NHANES study population reached control after 1 year follow-up [39]. Similarly, 40% of the treated hypertensive patients achieved their BP target in England after 1 year follow-up [14, 40]. Even though I-PREDICT design is different from NHANES and the European surveys’ one, early follow-up of BP may have contributed to better intervention thus leading to high rate of BP control in our population study.

BP outcomes according to JNC VII guidelines stratification was used throughout the analysis. The results of kappa statistics regarding the level of agreement between physicians’ perception and guidelines showed poor to fair agreement according to Landis and Koch’s classification. Such level of agreement reflects a gap between clinical practice and international guidelines. Factors related to insufficient access to guidelines at the point of care, insufficient information technology systems, especially for the small or solo practices, in addition to the culture, beliefs and habits of physicians and guidelines development have all been proposed as barriers to physician adherence to guidelines [41]. In fact, physicians tend to rely on their own judgment and personal experience to determine whether or not they are taking the right initiative for patients. Moreover, guidelines themselves often lack sufficient flexibility and relevance to clinical practice. Many guidelines do not reflect the complexity and context in which real clinical decisions must be made [41].

### Limitations

We acknowledge several limitations of the study. First, a sampling bias might have occurred at two levels: physicians’ selection and patients’ selection, which may affect the generalization of our results to the whole Lebanese population. Second, assessment of BP control was based on the measurement of diastolic and systolic BP at the clinic visit. People with hypertension often experience a spike in BP when the reading is taken in a physician’s office, leaving doctors with inaccurate information to determine the course of treatment and the status of BP control. To account for this “white coat effect”, researchers found significantly greater accuracy when several BP readings were combined from measurements taken at home or in the doctor’s office [42–44].

Finally, some studies have shown that advancing age is the foremost reported predictor factor of poor BP control [17, 24, 45, 46]. However, we are unable to retrieve this data for the time being.

## Conclusion

I-PREDICT survey has the merit to be the first national study involving a large number of centers across a developing country. The results of this survey are informative as they suggest early control of BP and combination therapy as predictive factors of better BP control, and diabetes type 1 and 2 as a predictor of poor BP control in the population. Patients with these characteristics may represent important factors to target for better control in clinical practice. Clearly, our study may contribute to closing the knowledge gap on the predictors for BP control in Lebanon. Another positive finding is that rate of BP control in our cohort was high compared to international studies. Nonetheless, several approaches are to be applied in order to improve physician adherence to clinical practice guidelines. Physicians can play a key role in implementing any comprehensive, national program to improve hypertension management in Lebanon, but their assessment should be more objective and based on guided criteria in order to avoid subjectivity and medical inertia [47, 48].

## Abbreviations

BMI: Body mass index
BP: Blood pressure
CI: Confidence interval
JNC: Joint National Committee
LICOR: Lebanese Interventional Coronary Registry
NHANES: National Health and Nutrition Examination Survey
OR: Odds ratio
WHO: World Health Organization.
